# Tyrosine 192 within the SH2 domain of the Src-protein tyrosine kinase p56^Lck^ regulates T-cell activation independently of Lck/CD45 interactions

**DOI:** 10.1186/s12964-020-00673-z

**Published:** 2020-11-23

**Authors:** Matthias Kästle, Camilla Merten, Roland Hartig, Thilo Kaehne, Ardiyanto Liaunardy-Jopeace, Nadine M. Woessner, Wolfgang W. Schamel, John James, Susana Minguet, Luca Simeoni, Burkhart Schraven

**Affiliations:** 1 Institute of Molecular and Clinical Immunology, Medical Faculty, Otto-von-Guericke University, Leipziger Str.44, Building 26, 39120 Magdeburg, Germany; 2 Health Campus Immunology, Infectiology and Inflammation (GC-I3), Medical Faculty, Otto-von-Guericke University, Magdeburg, Germany; 3 Institute of Experimental Internal Medicine, Medical Faculty, Otto-von-Guericke University, Magdeburg, Germany; 4 Molecular Immunity Unit, Department of Medicine, University of Cambridge, MRC-LMB, Cambridge, UK; 5 Spemann Graduate School of Biology and Medicine (SGBM), Albert-Ludwigs-University Freiburg, Freiburg, Germany; 6 Faculty of Biology, Signalling Research Centres BIOSS and CIBSS, University of Freiburg, Freiburg, Germany; 7 Center of Chronic Immunodeficiency CCI, University Clinics and Medical Faculty, Freiburg, Germany; 8 Division of Biomedical Sciences, Warwick Medical School, University of Warwick, Gibbet Hill, Coventry, UK

**Keywords:** Lck, T-cell activation, TCR signaling, Zap70, Knock-in mice, Y192, Signal transduction, PLA

## Abstract

**Background:**

Upon engagement of the T-cell receptor (TCR), the Src-family protein tyrosine kinase p56Lck phosphorylates components of the TCR (e.g. the TCRζ chains), thereby initiating T-cell activation. The enzymatic activity of Lck is primarily regulated via reversible and dynamic phosphorylation of two tyrosine residues, Y394 and Y505. Lck possesses an additional highly conserved tyrosine Y192, located within the SH2 domain, whose role in T-cell activation is not fully understood.

**Methods:**

Knock-in mice expressing a phospho-mimetic (Y192E) form of Lck were generated. Cellular and biochemical characterization was performed to elucidate the function of Y192 in primary T cells. HEK 293T and Jurkat T cells were used for in vitro studies.

**Results:**

Co-immunoprecipitation studies and biochemical analyses using T cells from Lck^Y192E^ knock-in mice revealed a diminished binding of Lck^Y192E^ to CD45 and a concomitant hyperphosphorylation of Y505, thus corroborating previous data obtained in Jurkat T cells. Surprisingly however, in vitro kinase assays showed that Lck^Y192E^ possesses a normal enzymatic activity in human and murine T cells. FLIM/FRET measurements employing an Lck^Y192E^ biosensor further indicated that the steady state conformation of the Lck^Y192E^ mutant is similar to Lck^wt^. These data suggest that Y192 might regulate Lck functions also independently from the Lck/CD45-association. Indeed, when Lck^Y192E^ was expressed in CD45^−/−^/Csk^−/−^ non-T cells (HEK 293T cells), phosphorylation of Y505 was similar to Lck^wt^, but Lck^Y192E^ still failed to optimally phosphorylate and activate the Lck downstream substrate ZAP70. Furthermore, Lck^Y19E^ was recruited less to CD3 after TCR stimulation.

**Conclusions:**

Taken together, phosphorylation of Y192 regulates Lck functions in T cells at least twofold, by preventing Lck association to CD45 and by modulating ligand-induced recruitment of Lck to the TCR.

**Major findings:**

Our data change the current view on the function of Y192 and suggest that Y192 also regulates Lck activity in a manner independent of Y505 phosphorylation.

**Video Abstract**

## Introduction

Lck, a member of the Src-family of tyrosine kinases, is primarily expressed in thymocytes and mature T cells [1, 2]. Lck initiates signaling events downstream of the TCR by phosphorylating tyrosine residues within the immunoreceptor tyrosine-based activation motifs (ITAMs) of the T cell receptor (TCR), which are present in the cytosolic tails of the TCR-associated CD3 and ζ chains (recently reviewed in [3]). ITAM phosphorylation by Lck is followed by recruitment of the Syk-family kinase ZAP70 to the activated TCR-complex. Upon subsequent phosphorylation and activation by Lck, ZAP70 further propagates TCR signaling by phosphorylating the transmembrane adapter protein linker for activation of T cells (LAT). The phosphorylation of LAT on multiple tyrosine residues allows the assembly of a central signalosome, which finally activates a number of intracellular signaling pathways leading to transcriptional activation and T-cell responses [4].

During the past decades, the importance of Lck in T-cell biology has become evident from a number of studies using both Lck-knockout mice and Lck-deficient T-cell lines. Lck^−/−^ mice display a marked thymic atrophy with 10% of normal cellularity [5, 6] and studies using an Lck-deficient Jurkat T-cell variant (J.Cam1.6) showed that induction of tyrosine phosphorylation and Ca^2+^ flux are strongly impaired upon TCR stimulation [7]. The signaling function of Lck in primary peripheral T cells has been elegantly assessed in transgenic mice in which the expression of Lck can be inducibly regulated [8]. These approaches have shown that peripheral Lck^−/−^ T cells are impaired in initiating proximal TCR signaling and display attenuated phosphorylation of TCRζ, ZAP70, LAT, PLC-γ1 as well as altered Ca^2+^ mobilization [9]. These signaling defects correlate with a reduced CD3-mediated proliferation and CD69 upregulation [9, 10].

The kinase activity of Lck is tightly controlled by different regulatory elements among which two well-characterized tyrosine residues, Y394 located in the activation loop within the kinase domain and Y505 at the C-terminus, as well as the SH2 and SH3 domains are of major importance [1, 11–15]. The currently accepted model proposes that Lck dynamically switches between a closed/inactive and an open/active conformation. Inactivation of Lck occurs upon intramolecular interactions between phosphorylated inhibitory Y505 and the SH2 domain and between the SH3 domain and a proline-rich region located within the SH2-catalytic domain linker [12]. Together these intramolecular interactions stabilize the closed and inactive conformation of Lck. Dephosphorylation of Y505 by the tyrosine phosphatase CD45 releases the closed conformation and induces the open/active conformation of Lck, which further requires the phosphorylation of Y394 [14, 16–18].

More recently, the view on Lck regulation has been expanded by new observations suggesting that Lck can adopt at least 4 different forms within T cells. Thus, in addition to the closed/inactive and the open/active form, a so called “primed” form, which is not phosphorylated on either Y394 or Y505, and a Y394/Y505-doubly phosphorylated form have been identified in primary human T cells and in T-cell lines [19]. Despite the fact that T cells express all four different forms of Lck, it has been proposed that the pool of constitutively active Lck (phosphorylated on Y394 alone or doubly phosphorylated on Y394 and Y505) is sufficient to initiate TCR signaling without additional dynamic changes in the conformations of Lck upon TCR stimulation [19, 20]. However, using an Lck biosensor combined with FLIM/FRET analyses, we have more recently shown that (1) a small fraction of Lck (about 20%) is de novo activated upon TCR stimulation and (2) that the TCR-mediated opening/de novo activation of Lck is required for initiation of TCR-mediated signaling processes [18, 21]. In addition, we found that, despite being in an open conformation, the non-phosphorylated and primed form of Lck is not catalytically active. This observation implied that de novo phosphorylation of Y394 is mandatory for the initiation of TCR signaling [18], an assumption that has meanwhile been corroborated by independent investigations [17].

**Fig. 1:**
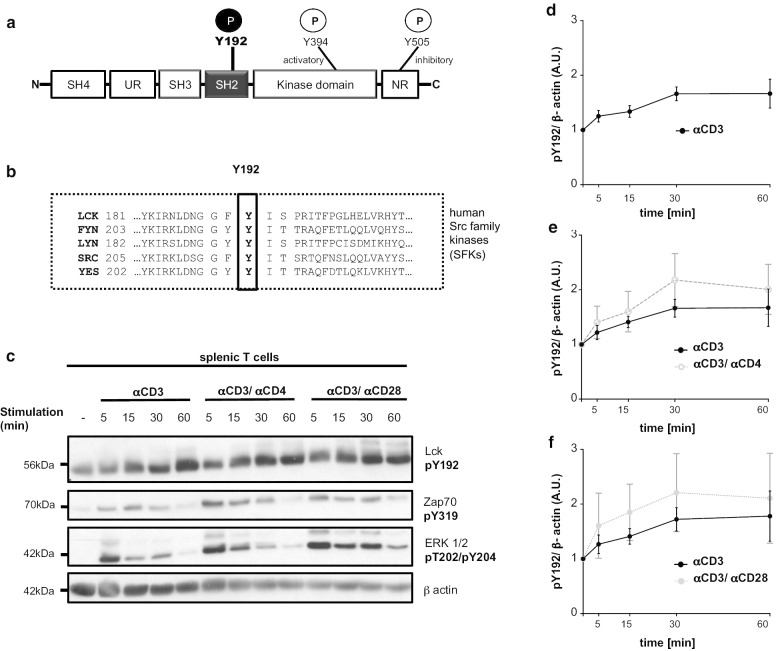
Lck^Y192^ is inducibly phosphorylated upon TCR stimulation in splenic murine T cells. **a** Cartoon showing the structure of Lck including the regulatory domains and tyrosine phosphorylation sites. **b** Lck-Y192 is conserved among Src-family kinases. The amino acid sequences of representative human Src-family kinases are shown. **c** Murine splenic T cells were stimulated with a CD3 antibody immobilized on microbeads for the indicated periods of time. Co-stimulation occurred with either CD4 or CD28 antibodies. Subsequently, cells were lysed and immunoblot analyses were carried out using a pY192-phosphospecific antibody to monitor phosphorylation dynamics of Y192. The efficiency of T-cell activation was measured using phospho-Zap70 and phospho-Erk1/2 antibodies, respectively. Equal protein loading was verified using a β-actin antibody. PhosphoY192 signals from splenic T cells stimulated with CD3 alone (n = 5) (**d**), CD3/CD4 (n = 4) (**e**), or CD3/CD28 (n = 3) (**f**) were normalized to β-actin and quantified. For the densiometric analysis the median of the Western blot bands were taken. Mean values ± SEM of the indicated experiments are shown

In addition to Y394 and Y505, a third highly conserved regulatory tyrosine Y192, lying within the SH2 domain (Fig. 1a, b), has been proposed to regulate the signaling function of Lck. The SH2 domain of Lck is one of the crucial regulatory elements as it is not only involved in the intra-molecular interaction with phosphorylated Y505 but also in a number of inter-molecular interactions including ZAP70 [22], Shp1 [23], and TSAd [24], all of which are assumed to be important regulators of Lck. It has been suggested that phosphorylation of Y192 may regulate Lck and TCR signaling by altering the ligand specificity of the SH2 domain [24, 25]. Recent studies have proposed a model in which phosphorylation of Y192 by ZAP70 triggers a negative feedback loop [26] that involves the protein tyrosine phosphatase CD45 [27]. The failure of pY192 to interact with CD45 leads to hyperphosphorylation of Y505, thereby inducing an inactivation of Lck and impaired TCR signaling. This model was primarily based on data that were obtained in an Lck-deficient variant of the Jurkat T-cell line (J.Lck) [27]. Currently, it is unclear whether a similar mechanism also operates in primary T cells.

To study the function of Y192 in vivo, we generated Lck^Y192F^ and Lck^Y192E^ knock-in mice. In line with Courtney et al., we here show that primary murine peripheral T cells expressing the Lck^Y192E^ mutant display a strong hyperphosphorylation on Y505 and an altered association between Lck^Y192E^ and CD45 [27]. In addition, Lck^Y192E^ expressing T cells are impaired in TCR-mediated signaling and CD3-mediated proliferation. Surprisingly however, in vitro kinase assays revealed that the Lck^Y192E^-mutant possesses the same enzymatic activity as wild type Lck (Lck^wt^). In line with this, FLIM/FRET measurements showed that under steady state conditions, namely in the absence of TCR-mediated stimuli, the conformation of the Lck^Y192E^ mutant is comparable to Lck^wt^. In order to elucidate whether Y192 might regulate Lck function independently of the phosphorylation of Y505, Lck^Y192E^ was expressed in two different variants of HEK 293T cells expressing or not the TCR-CD3 complex. These cells lack CD45 and Csk expression and Lck^Y192E^ did not show hyperphosphorylation of Y505. Still, Lck^Y192E^ even in these cells was unable to fully phosphorylate and activate ZAP70. Recently, it was shown that recruitment of Lck to the ligand-bound TCR promotes its phosphorylation thereby locally increasing Lck activity [28]. Proximity ligation assays (PLA) showed that the recruitment of the Lck^Y192E^ mutant to the TCR was attenuated upon TCR stimulation. This suggests that the conformational change of Lck, that is required to initiate membrane proximal signaling in T cells, is attenuated in cells expressing Lck^Y192E^.

In summary, while our data to a large extent corroborate previous findings made in Jurkat T cells, they challenge the view that impaired signaling in T cells expressing Lck^Y192E^ is exclusively due to altered enzymatic activity of Lck mediated by loss of Lck/CD45 interactions.

## Material and methods

### Antibodies and reagents

1. FACS antibodies

**Table:** 

Antibody	Clone	Company
CD4 FITC	GK 1.5	Biolegend, BD Bioscience
CD8 PE	53-6.7	Biolegend, BD Bioscience
CD3 APC	145-2C11	BD Bioscience
B220 FITC	RA3-6B2	BD Bioscience

2. Western blot antibodies

**Table:** 

Antibody	Clone/Lot	Company
Lck	3A5	Santa Cruz
Lck	06-583	Merck Millipore, upstate
pLck-Y505	2751P	Cell Signaling Technology
pSrc 416	2101S	Cell Signaling Technology
ZAP70	1E7.2	Santa Cruz
pZAP70	2701S	Cell Signaling Technology
PLCγ	05-163	Merck Millipore
pPLCγ	2821S	Cell Signaling Technology
Lat	11B.12	Santa Cruz
pLat	3584S	Cell Signaling Technology
pTyr	4G10	–
β-actin	A5441	Sigma
pLck Y192	LS-C199194-50	Biozol
p-p44/42 MAPK (T202/204)	9101S	Cell Signaling Technology

3. ELISA antibodies

**Table:** 

Antibody	Clone/Lot	Company
CD45	30-F11	Biolegend
Anti-mouse-AP	A9316	Sigma Aldrich

4. Stimulation antibodies

**Table:** 

Antibody	Clone	Company
CD3ε	UCHT1	Ebioscience, Biolegend
CD3ε biotin	145-2C11	Ebioscience, BD Pharmingen, Biolegend
Idiotypic TCR	C305	–
CD4	GK 1.5	BD
CD4	RM4-5	Biolegend
CD28	37.51	Biolegend

## Experimental models

### Cells

Cells were maintained at 37 °C with 5% CO_2_. JE6 (human leukemic T-cell line) were cultured in RPMI 1640 (Roswell Park Memorial Institute) supplemented with 10% FBS (fetal bovine serum) and 1% penicillin/streptomycin. For this study, JE6 and the Lck-deficient T-cell lines (J.Lck, [27]) were used. HEK 293T cells (human embryonic kidney 293T cells) were cultured in DMEM (Dulbecco's modified Eagle's medium) with 10% FBS and 1% penicillin/streptomycin. To generate and maintain stable cell lines, the antibiotic puromycin was added to the supplemented medium. The CRISPR/Cas Lck deficient Jurkat T cell line (J.Lck) was kindley provided by Prof. Dr. Arthur Weis (University of Calfornia). Dr. John James (University Warwick) provided the HEK 293T + TCR/CD3 cells [17, 29].

### Plasmids

In this study the following plasmids were used:

**Table:** 

Vector	Supplier	Transfected cell lines
pEF-Lck-IRES-GFP	Vectorbuilder	J.Lck
PB pEF-Lck_CMV-GFP/Puro	Vectorbuilder	HEK293T,HEK293T-TCR
pEF-TqLckV-2	[18]	J.Lck
pMyc- ZAP70	[30]	HEK293T, HEK293T-TCR
pEF_hyPBase	Vectorbuilder	HEK293T, HEK293T-TCR
pHR-Lck* vector	[17]	HEK293T-TCR
PB pEF-Lck_T2A_GFP	Vectorbuilder	J.Lck
PB_pEF-Lck-biosensor (5^th^ generation)	Vectorbuilder	J.Lck

### Site-directed mutagenesis

To mutate the Lck tyrosine (Y) 192 to glutamic acid (E), the Agilent Quick Change II XL (Agilent) system was used according to the manufacturer’s instructions. The mutation was inserted into the plasmids mentioned above. The Lck Y192E primers were designed using the tools from Agilent and synthesized by Biomers. The following primers were used:

**Table:** 

Lck Y192E fw	5′ gga caa cgg tgg ctt cga gat ctc ccc tcg aat cac 3′
Lck Y192E rev	5′ gtg att cga ggg gag atc tcg aag cca ccg ttg tcc 3′

### Cell transfections

DNA electroporation of Jurkat T-cell lines was performed using the Gene Pulser II System (BIORAD) as previously described [30]. For the FLIM/FRET measurements, cells were cultured in RPMI 1640 without phenol red (Gibco). HEK 293T and HEK 293T–TCR/CD3 cells were transfected as described in [30].

### Generation of stable cell lines

To generate stable cell lines, the PiggyBac transposon system was used (Vectorbuilder, [31]). HEK 293T cells were transfected with 1 µg of a Piggy bac plasmid (PB pEF-Lck_CMV-GFP/Puro) and 0.6 μg of a hyperactive transposase (pEF_hyPBase) as described above. The transfected cells were cultured in DMEM medium supplemented with 10% FBS, 1% pennicillin/streptomycin and 0.5 μg/ml puromycin (Gibco) s a selection marker. Jurkat T cells were electroporated with one of the Piggy bac plasmids (PB pEF-Lck_T2A_GFP, PB pEF-Lck-biosensor) and a hyperactive transposase (pEF_hyPBase) as described above. Transfected Jurkat T cells were sorted with the Aria Cell Sorter 3 (BD Bioscience) and afterwards maintained in RPMI supplemented with 10% FBS, 1% pennicillin/streptomycin and 0.1% Ciprobay.

## Mice

Mice were kept in a pathogen-free facility at the Medical Campus of the University of Magdeburg according to the German animal law. Lck knock-in mice (Lck^Y192E^, Lck^Y192F^) were generated by Prof. Marco Herold and Dr. Andrew Kueh (WEHI, Melbourne) using CRISPR/Cas technology. The obtained heterozygous mice were backcrossed to a C57BL/6JRJ (Janvier Labs) genetic background.

The animals were genotyped by PCR and sequencing using the following primers (Biomers):

**Table:** 

Lck Y192 fw	5′ tcagggtccttttccctgtc 3′
Lck Y192 rev	5′ ctgaaggggaatgaaagacg 3′

The sequencing was performed by Dr. Denny Schanze (Institute of Human Genetics, University of Magdeburg).

### Isolation of murine lymphatic organs and immune cells

Cells from thymus, spleen, and lymph nodes were isolated using a 70 µm cell strainer (Falcon). Splenic T cells were isolated using a pan T-cell isolation kit and AutoMACS separator from Miltenyi Biotech.

### Stimulation and lysis of cells

2.5*10^6^ to 5*10^6^ murine thymocytes and splenic T cells were stimulated with 5–10 µg/ml biotinylated 145-2C11 (Biolegend) followed by cross-linking with 20 µg/ml neutravidin at 37 °C. Alternatively, cells were stimulated with biotinylated CD3ε Ab immobilized on superavidin-coated microbeads (Bang Laboratories Inc) as previously described [32]. Costimulation occurred via biotinylated CD3/CD28 (Biolegend) or CD3/CD4 (Biolegend) immobilized on superavidin-coated microbeads. 1*10^6^ Jurkat T cells were stimulated with anti-idiotypic TCR antibody (C305). Cells were lysed in 1% LM (an *N*-dodecyl b-maltoside), 1% NP-40, 1 mM phenylmethylsulfonyl fluoride, 10 mM NaF, 10 mM EDTA, 50 mM Tris–HCl (pH 7.5), and 150 mM NaCl.

### Immunoblotting

Samples were assayed using SDS-PAGE. Proteins were transferred (semi-dry) on a polyvinylidene difluoride membrane (Amersham). Membranes were blocked in 5% milk and incubated with primary antibodies in 5% milk or 5% BSA for 1 h. Secondary antibodies coupled with a fluorophore (LI-COR) or horseradish phosphates (Dianova) were diluted in 5% milk. To detect the protein signals on the membranes, an Odyssey infrared imager (LI-COR) and ECL (Amershan) were used. The densiometric analyses of the blots were performed with the image software Image Studio (LI-COR). The total median values of densiometric analysis were used for quantifications.

### Immunoprecipitation and in vitro kinase assay

Lck was immunoprecipitated using a polyclonal antibody (06–583; Merck Millipore) and Protein A Agarose beads (Santa Cruz Biotechnology) for 2 h at 4 °C as previously described [21]. To remove unspecific bindings, the immunoprecipitates were washed in washing buffer (1% LM, 1% NP-40, 50 mM Tris–HCl, 165 mM NaCl, NaF and 1% PMSF) five times and were divided in two parts. One part (50%) of the samples was assayed for Lck expression using immunoblotting. The rest of the immunoprecipitates was assayed for in vitro kinase activity. Briefly, samples were resuspended in kinase buffer (20 mM Tris–HCl (pH 7.5), 100 mM ATP, 10 µCi of 32P-ATP) and incubated for 20 min at 30 °C. The immunoprecipitates were washed four times in washing buffer (20 mM Tris–HCl (pH 7.5), 20 mM EDTA, 150 mM) and the kinase activity of Lck was investigated using SDS-PAGE and autoradiography.

### ELISA/CD45 phosphatase assay

To assess CD45/Lck interaction in thymocytes, an ELISA and phosphates assay were used and modified from Schraven et al. [33]. Nunc 96 well plates (Thermo Fisher Scientific) were coated with mouse CD45 antibody (30 F-11, Biolegend) in PBS (1:100) overnight. For each control and condition 6 wells were used. Between all steps, the plates were washed with PBS + 1% BSA. The coated wells were blocked with PBS + 10% BSA for 1 h. Isolated thymocytes were lysed for 20 min at 4 °C with a buffer containing 1% Brij 58, 150 mM NaCl, 150 mM Tris–HCl, 1% PMSF, 1%, Protease Inhibitor mix (Merck) and were added to the plates to immunoprecipitate CD45 at 4 °C overnight. 10% of the lysates were used for Western blotting analysis to assess total Lck expression with a monoclonal antibody (Lck, 3A5, Santa Cruz). All six CD45 immunoprecipitates were washed four times with washing buffer (1% Brij 58, 165 mM NaCl, 50 mM Tris–HCl, 1% PMSF). To verify the immunoprecipitation of CD45, three of the six wells of each condition were treated with 2.5 mM pNPP substrate (New England Biolabs) in 50 mM Hepes; 100 mM KCl, 19 mM DTT, 0,1% Triton X100 and CD45 phosphatase activity was measured after 4–10 h with a Tecan Safire reader and the Tecan Magellan software (Tecan lifesciences). To analyze CD45/Lck interaction, the 3 remaining CD45 immunoprecipitates were incubated with an Lck (1:1000) antibody diluted in PBS + BSA for 1 h and anti-mouse (1:30,000) coupled with alkaline phosphatase (Sigma Aldrich). The substrate (MAB tech) was added and the samples were measured with the Tecan Safire reader at 15, 30, 60 and 120 min. To analyze the interaction between CD45 and Lck, the amount of the Lck expression in the input lysates was analyzed densiometrically with Image Studio (LI-Cor). The ratio between the OD values of CD45-associated Lck and the Lck input assessed by densitometrical analyses was used to quantify CD45/Lck association. The phosphatase assay was used to verify that the CD45 immunoprecipitaiton was successful.

### FACS measurements

Cell suspensions from thymus, spleen and lymph nodes were prepared. The antibodies (BD Bioscience or Biolegend) were diluted 1:100 in PBS and for each staining and added to the cells (1*10^6^ cells/sample). Samples were measured with the BD Fortessa I (3 Lasers) and the BD Calibur using the BD FACSDiva Software and BD CellQuest (BD Bioscience). The data were analyzed using FlowJo software (BD Bioscience).

### Calcium flux

Isolated splenic T cells were loaded with Indo-1 (Thermo Fisher Scientific) for 45 min at 37 °C in RPMI 1640 without phenol red (Gibco) and were washed with RPMI without phenol red for 45 min at 37 °C. Stimulation was induced by the addition of CD3ε (145-2C11; Biolegend) and CD4 (GK1.5; BD Bioscience) antibodies followed by neutravidin cross-linking. As a positive control, ionomycin (10 mg/ml Sigma-Aldrich) was added 8 min after Ab stimulation. Calcium influx was measured with a LSR I analyzer (BD Bioscience) as described in [21] using a 325 nm laser line of a helium cadmium laser. The emission wavelength ranges from 390 to 420 nm and from 500 to 520 nm were detected and the ratio of the two emission intensities was calculated and analyzed with FlowJo (BD Bioscience).

### Proliferation

Splenic T cells (50 000 cells/well) were cultured in RPMI 1640 medium (supplemented with 10% FCS, antibiotics, 2-ME) in 96-well plates (Costar) in the presence of plate-bound CD3ε antibody (1 μg, 145-2C11; BD Biosciences or Biolegend) or PMA/Ionomycin as a positive control. After 72 h the cultured cells were labeled with 1 μCi [^3^H] thymidine per well during the last 8 h.

### Uncaging of Lck by illumination

The experiments to photocage Lck to quantify the kinase phosphorylation kinetics were performed essentially as described in [17]. Briefly, the Y192E mutation was first introduced into the pHR-Lck* vector, which also has Lck K273 mutated to the UAG stop codon and is fused to eGFP. pHR-Lck* was then transfected into HEK 293T cells expressing the complete TCR complex, along with ZAP70 and the plasmids required for the incorporation of photo-caged Lysine (pc-Lys) into the active site. After 48 h, transfected cells were resuspended and uncaged by global UV illumination to initiate Lck kinase activity, measured by the phosphorylation of ZAP70 at Y319. Flash-freezing was used to rapidly quench the reaction at defined time points and the intensity of ZAP70 phosphorylation was quantified by Western blot analysis. The experiment was repeated three times to quantify the kinase activity of Lck^Y192E^ compared to Lck^wt^ and Lck^Y394^ controls.

### Localization studies/fluorescence microscopy

J.Lck transfected with either Lck^wt^ or Lck^Y192E^ were washed two times and were resuspended in PBS. 3*10^4^ cells per condition were used on the microscope slide (Marienfeld Gmbh). The cells on the slide were fixed in 1% PFA and 0,025% glutaraldehyde for 15 min. The permeabilization of the cell membrane was performed with 0.2% Triton X100 for 10 min and the cells were blocked in 1% BSA for 30 min. Staining was performed with a 1:50 diluted Lck antibody (clone 3A5) (Santa Cruz) for one hour. As a secondary antibody, a goat anti-mouse conjugated with an Alexa Fluor647 was used. The pictures were captured with a SP8 confocal microscope (Leica).

### FLIM/FRET measurements

To study the conformation of the Lck^Y192E^ mutant, a previously described Lck biosensor was used [18]. Lck-deficient Jurkat T cells (J.Lck) were transfected with mTurquoise as a negative FRET control and the different Lck-biosensor mutants. The FRET signal was analyzed via the fluorescence mean lifetime as described in [18, 21, 34]. To study conformational dynamics upon TCR stimulation, J.Lck stably expressing a modified Lck biosensor (5^th^ generation) were used. In this newly modified Lck biosensor, an optimized FRET donor mTurquoise is positioned in front of the SH3 domain and does not contain any linker region between the fluorophores and the Lck backbone. Changing the position of mTurquoise resulted in a more pronounced conformational change upon stimulation. The experiment setup and the analysis workflow were performed as described in [18]. The arithmetic mean of all the cells was calculated using GraphPad Prism.

### PLA

Each sample of 0.9*10^5^ cells was starved and rested on diagnostic microscope slides (Thermo Fisher Scientific) at 37 °C for 1 h. Cells were treated with the anti-idiotypic TCR antibody C305 (1:50) or with 1 mM pervanadate (PerV) for 5 min at 37 °C. Cells were then fixed with 2% PFA for 15 min at room temperature, permeabilized with 0.5% saponin for 30 min and blocked. Blocked cells were stained according to the manufacturer’s instructions with the Duolink kit (Olink Bioscience). The antibodies used were goat CD3ε (1:600, EB12592, Everest Biotech) and mouse Lck (1:200, 3A5, Cell Signaling). Nuclei were stained with DAPI (Roth). Images were taken at 60× magnification with a confocal microscope (Nikon C2) and analyzed with BlobFinder. The PLA conditions were set to ensure that the total number of blobs per image analyzed was kept under 10^3^ to provide accurate counting of the number of blobs per cell as previously suggested by the developers of BlobFinder.

### Statistics

Statistical analyses were performed using GraphPad Prism software. Unless otherwise indicated, statistical significance was determined between groups using an unpaired Student's *t*-test. The minimum acceptable level of significance was *P* < 0.05.

## Results

### Kinetics of Lck^Y192^ phosphorylation upon TCR stimulation in primary murine T cells

In Jurkat T cells, it has previously been shown that Lck^Y192^ is constitutively phosphorylated and that TCR stimulation induces a rapid increase in the Y192 phosphorylation [25, 26, 35–37]. Since the phosphorylation status of Lck^Y192^ in primary T cells is unknown, we monitored the dynamics of Y192 phosphorylation upon T-cell activation in splenic murine T cells using a phospho-Y192 specific antibody. As shown in Fig. 1c, d, splenic T cells display a constitutive phosphorylation on Y192, which increases upon TCR stimulation. Compared to CD3-stimulation alone, co-stimulation via either the CD4 or CD28 co-receptors further augments the phosphorylation of Y192 (Fig. 1c, e, f). Hence, in contrast to Y394 (and Y505), whose phosphorylation status does not change upon TCR/CD3-stimulation alone [19, 21], the phosphorylation status of Y192 appears to be regulated by both the TCR/CD3-complex and by coreceptors and costimulatory molecules such as CD4 or CD28, respectively. In addition, the augmented phosphorylation of Y192 upon T-cell activation suggests that dynamic changes in the phosphorylation status of Y192 may influence the function and/or activity of Lck, thereby regulating TCR-mediated signaling processes in thymocytes and peripheral T cells.

### Defective T-cell development in Lck^Y192E^ mice

To assess the function of Y192 in vivo, we generated non-phosphorylatable Lck^Y192F^ and phosphomimetic Lck^Y192E^ knock-in lines using the CRISPR/Cas9 technology. We first investigated T-cell development in the two different mouse strains. Flow cytometry analysis revealed that the distribution of the main thymic T-cell subsets (as defined by the expression of CD4 and CD8) of Lck^Y192F^ mice were comparable to that of Lck^wt^ controls (Additional file 1: Fig. S1A, B), thus indicating that T-cell development is not affected by the Lck^Y192F^ mutation. In agreement with the normal T-cell development, also peripheral T cells were unaffected in Lck^Y192F^ mice (Additional file 1: Fig. S1C). Similarly, CD3-mediated global tyrosine-phosphorylation was unaltered in Lck^Y192F^ splenic T cells (Additional file 1: Fig. S1D). Collectively, the analyses of the Lck^Y192F^ knock-in animals corroborate previous findings made by Courtney et al. in Jurkat T cells [26].

**Fig. 2:**
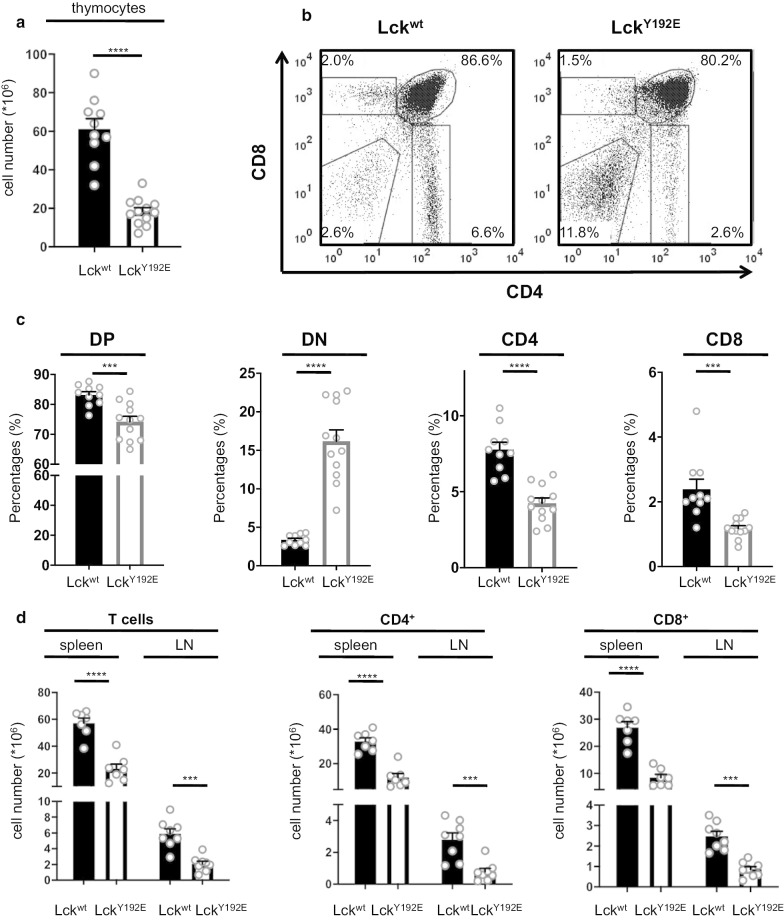
Block of T-cell development in knock-in mice expressing the phosphomimetic Lck^Y192E^ mutant. **a** Total numbers of thymocytes obtained from Lck^wt^ and Lck^Y192E^ knock-in mice. **b** Thymic subpopulations were identified upon staining with CD4 and CD8 antibodies by flow cytometry. **c** Percentages of the thymocyte subpopulations of several experiments are shown. **d** Numbers of total peripheral T cells and of peripheral CD4^+^ and CD8^+^ T cell subsets. Each dot represents one mouse. Mean values of all characterized mice + SEM are represented. Statistical analyses were performed using an unpaired Student’s t test, ****p < 0.0001; ***p < 0.001

In striking contrast to Lck^Y192F^ mice, the Lck^Y192E^ mice displayed a strong decrease in total thymocyte numbers (Fig. 2a) and severe alterations in the distribution of thymic subsets (Fig. 2b, c). Consistent with the thymic defect, total T-cell numbers as well as the numbers of both CD4^+^ and CD8^+^ peripheral T cells were strongly decreased in the spleen and lymph nodes of Lck^Y192E^ animals (Fig. 2d). In particular, the fraction of both naïve CD44^low^CD4^+^ and CD44^low^CD8^+^ T-cell subsets were reduced in the lymph nodes of Lck^Y192E^ mice, whereas the fractions of antigen-experienced and memory-like (defined as CD44^high^) T cells remained unchanged or slightly increased (Additional file 1: Fig. S2A). Interestingly, CD3 expression was reduced in both CD4^+^ and CD8^+^ Lck^Y192E^ T cells (Additional file 1: Fig. S2B), thus suggesting that only thymocytes expressing low TCR levels are positively selected in the Lck^Y192E^ knock-in mice. Conversely to T cells, the number of B cells was largely unaffected (Fig S2C). Thus, the developmental defect observed in the thymus results in strongly reduced numbers of naïve T-lymphocytes in the periphery of Lck^Y192E^ mice. Collectively, our data demonstrate that Y192 plays an important function in the regulation of thymic development and hence in the generation of mature T cells.

### Impaired response to TCR stimulation in Lck^Y192E^ T cells

**Fig. 3:**
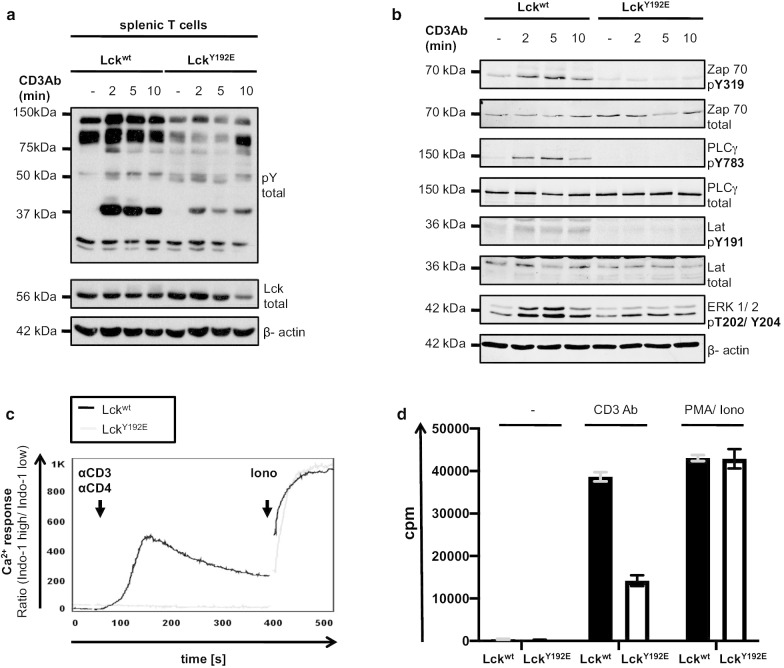
Impaired TCR signaling in peripheral T cells from Lck^Y192E^ knock-in mice. **a** Splenic T cells from Lck^wt^ and Lck^Y192E^ knock-in mice were stimulated with a CD3 antibody. At the indicated time points after stimulation, cells were lysed and the levels of global protein tyrosine phosphorylation were assessed using a pan phosphotyrosine antibody (pY total). One representative experiment is shown (n = 4). **b** The activation/phosphorylation of selected and important signaling molecules in T cells was investigated using the indicated phosphospecific antibodies. Equal protein loading was verified using antibodies directed against corresponding total proteins or against β-actin, respectively. **c** Calcium influx in peripheral T cells was assessed upon CD3xCD4 cross-linking by flow cytometry. The Ca^2+^ flux was calculated via the ratio of Indo-1 high/Indo-1 low expressing cells. Ionomycin was used to show equal loading with Indo-1. One representative of three independent experiments is shown. **d** Proliferation of splenic T cells was assessed using [3H]-thymidine incorporation upon stimulation with a CD3 antibody (1 µg). PMA + ionomycin were used as positive control. One representative of four independent experiments is shown

We next investigated the signaling phenotype of the peripheral T cells obtained from Lck^Y192E^ animals. As shown in Fig. 3a–c, peripheral Lck^Y192E^ T cells displayed strongly impaired TCR-mediated signaling at both the proximal and distal levels. Besides a failure in TCR-mediated induction of global tyrosine phosphorylation (Fig. 3a), phosphorylation and activation of important signaling molecules (Fig. 3b) and induction of calcium influx (Fig. 3c), we also observed that T cells expressing Lck^Y192E^ did not efficiently proliferate in response to CD3 stimulation (Fig. 3d). Hence, peripheral T cells carrying the Lck^Y192E^ mutation appear to be largely signaling incompetent. We corroborated the findings made in primary murine Lck^Y192E^ T cells in Jurkat T cells stably expressing the Lck^Y192E^ mutant. Indeed, Lck-deficient J.Lck cells reconstituted with Lck^Y192E^ showed impaired TCR-induced global tyrosine phosphorylation and reduced phosphorylation of ZAP70 (Additional file 1: Fig. S3A) despite the fact that CD3 expression was comparable (Additional file 1: Fig. S3B) and Lck expression even considerably higher than in J.Lck cells expressing Lck^wt^ (Additional file 1: Fig. S3A). Together these data indicate that peripheral murine T lymphocytes and Jurkat T cells expressing Lck^Y192E^ are largely signaling incompetent, in agreement with a previous report [27].

### Lck^Y192E^ from primary murine T cells displays hyperphosphorylation on Y505 and diminished binding to CD45

**Fig. 4:**
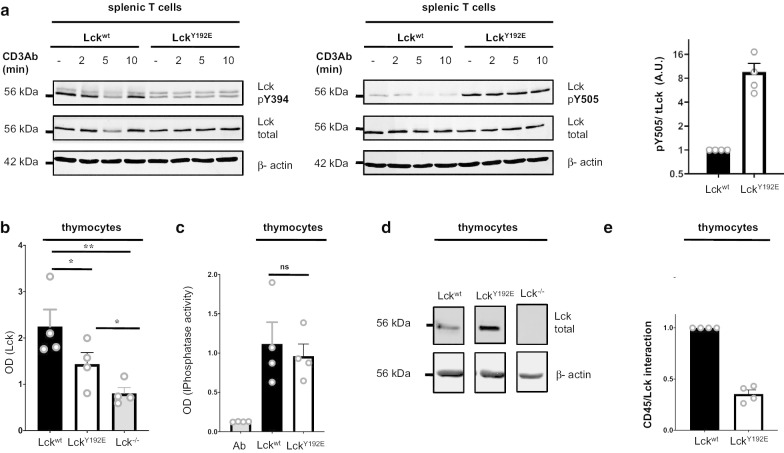
Hyperphosphorylation of Y505 in Lck^Y192E^ and decreased Lck^Y192E^/CD45 interaction. **a** Splenic T cells from Lck^wt^ and Lck^Y192E^ knock-in mice were stimulated for the indicated periods of time. Cells were subsequently lysed and the phosphorylation of Y394 and Y505 assessed using phosphospecific antibodies. The bar graph (right) shows the quantification of Lck phosphoryation on Y505 from four independent experiments. **b** Murine thymocytes were lysed and CD45 was immunoprecipitated in a 96-well plate. CD45-associated Lck was detected using an Lck antibody and a secondary alkaline phosphatase-conjugated Ab in a Tecan plate-reader. Thymocytes from Lck^−/−^ mice were used as negative control. **c** The amount of CD45 immunoprecipitated in **b** was assessed using a phosphatase assay. Ab represents the antibody control. **d** The amount of Lck in the input lysates used in **b** was assessed using an immunoblot. **e** Data in **b** were normalized against Lck expression shown in **d** to quantify CD45/Lck association. Each dot represents one experiment (n = 4). Statistical analyses were performed using a paired Student’s t test, **p < 0.01; *p < 0.05, *ns* not statistically significant

**Fig. 5:**
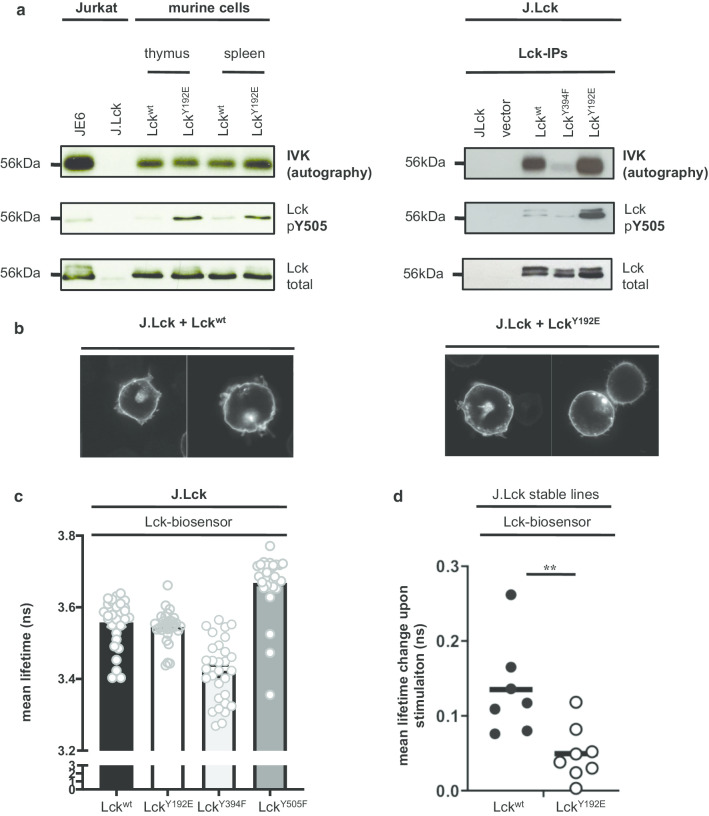
Lck^Y192E^ is catalytically active and in a conformation like Lck^wt^. **a** Thymocytes and splenic T cells from Lck^wt^ and Lck^Y192E^ knock-in mice (left) or J.Lck cells reconstituted with the indicated Lck constructs (right) were lysed and Lck was immunoprecipitated. Immunoprecipitaes were incubated with [^32^P] ATP and proteins were subsequently separated by SDS-PAGE. The activity of Lck was monitored by autoradiography, whereas the expression of Lck and the phosphorylation levels of Y505 were analyzed by immunoblotting. Lck immunoprecipitates from JE6 and J.Lck in the left panel were use as positive and negative control, respectively. Catalytically inactive Lck^Y394F^ in the right panel was used as negative control. One representative of two independent experiments is shown. **b** J.Lck expressing either Lck^wt^ or Lck^Y192E^ were labeled with an Lck antibody. Pictures were taken using a confocal microscope. The left panel show the subcellular localization of Lck^wt^, while the right panel covers Lck^Y192E^. **c** Lck-deficient J.Lck T cells were reconstituted with the indicated Lck-biosensor constructs. Graphs show mean lifetime of FLIM/FRET analyses. The constitutively closed (Y394F) and constitutively open (Y505F) Lck mutants served as controls as reported previously [18, 20, 21]. Dots represent individual cells from 3 experiments and the arithmetic mean ± SEM was calculated. **d** Lck-deficient Jurkat cells (J.Lck) stably expressing either a Lck^WT^ biosensor or a Lck biosensor carrying the Y192E mutation were used for dynamic FLIM/FRET measurements as previously described [18, 21]. Change in mean lifetime upon CD3 stimulation was calculated from 7 to 8 cells from two independent experiments (n = 2). Horizontal bar represents the mean, which was 0.135 ns for Lck^WT^ and 0.049 ns for Lck^Y192E^. Each dot represents one cell. Statistical analyses were performed using an unpaired Student’s t test **p < 0.01

It had been suggested that the inability of Lck^Y192E^ to initiate TCR signaling is mediated via an altered association between the Lck mutant and the protein tyrosine phosphatase CD45 [27]. In the proposed model, loss of Lck^Y192E^/CD45 interaction causes a hyperphosphorylation of Y505 that inactivates Lck, thereby abrogating TCR-mediated signaling. We assessed the tyrosine phosphorylation status of Lck^Y192E^ in T cells obtained from Lck^Y192E^ knock-in mice using phosphospecific antibodies directed at the two regulatory tyrosine residues, Y394 and Y505. Figure 4a shows that, similar to Jurkat T cells expressing the Lck^Y192E^ mutant ([27] and Fig. 5a), T cells of Lck^Y192E^ animals display a strong hyperphosphorylation of the negative regulatory tyrosine residue Y505 and a lower phosphorylation of Y394 compared to cells expressing Lck^wt^. Furthermore, analysis of CD45 immunoprecipitations obtained from Lck^wt^ and Lck^Y192E^ thymocytes revealed that also in primary cells the Lck^Y192E^ mutant shows a strongly reduced capability to associate with CD45 (Fig. 4b–e). Hence, the data obtained from Lck^Y192E^ expressing murine T cells are in line with previous findings in the Jurkat T-cell line [27].

### Lck^Y192E^ kinase activity and conformation are comparable to Lck^wt^

Loss of Lck/CD45 interaction and Y505 hyperphosphorylation of the Lck^Y192E^ mutant suggested that Lck^Y192E^ assumes the closed and inactive conformation. We analyzed the enzymatic activity of Lck^Y192E^ using a sensitive in vitro kinase asssay. To this end, we prepared Lck immunoprecipitates from J.Lck cells expressing either Lck^wt^ or Lck^Y192E^, or from both thymocytes and splenic T cells obtained from Lck^wt^ or Lck^Y192E^ knock-in mice. The immunoprecipitates were subsequently subjected to a classical in vitro kinase assay followed by SDS-PAGE and autoradiography. Surprisingly, Lck^Y192E^ showed the same (or even slightly increased) enzymatic activity as Lck^wt^ in both human and mouse T cells despite hyperphosphorylation of Y505 (Fig. 5a) and an unaltered subcellular distribution (Fig. 5b). These data indicated that the impaired proximal signaling in T cells expressing Lck^Y192E^ might not be exclusively due to the hyperphosphorylation of Y505. In line with the in vitro kinase data, we found that a FLIM/FRET-based Lck^Y192E^ biosensor assumes the same conformation as Lck^wt^ when expressed in Lck-deficient J.Lck cells under steady state conditions (Fig. 5c). Hence, despite hyperphosphorylation of Y505, the Lck^Y192E^ mutant displays the same enzymatic activity and conformation as Lck^wt^. The Lck biosensor is also capable of monitoring de novo activation and opening of Lck in response to CD3-mediated signals [18, 21]. We thus next aimed at assessing TCR-mediated changes in FRET using J.Lck cells either stably expressing an Lck^wt^- or an Lck^Y192E^-biosensor. The Lck^Y192E^-biosensor showed weaker changes of the FRET signal upon T-cell activation compared to the Lck^wt^-biosensor (Fig. 5d), suggesting that the Lck^Y192E^ mutant might lack the flexibility needed to be properly activated upon T-cell activation.

### The inability of Lck^Y192E^ to activate its substrate Zap70 is independent from the phosphorylation status of Y505

**Fig. 6:**
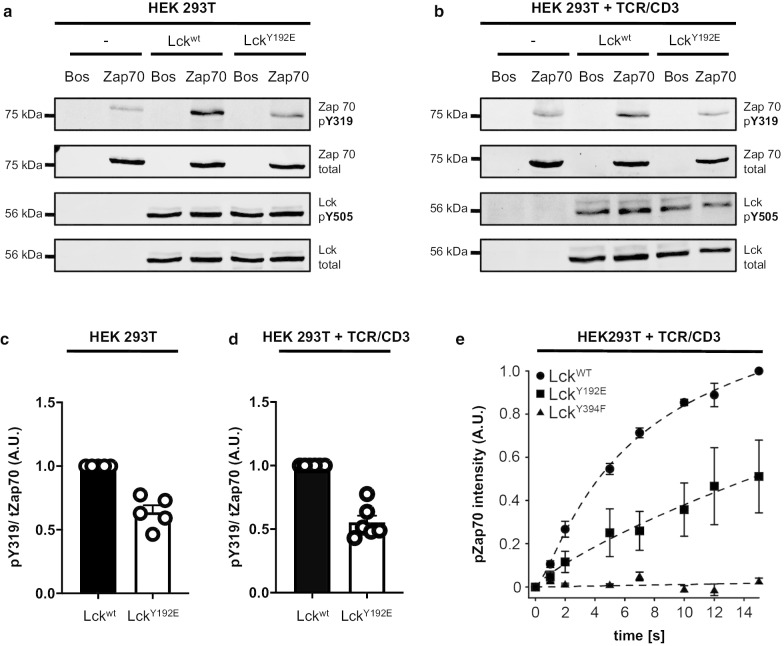
Lck^Y192E^ displays an impaired ability to phosphorylate ZAP70 independently of Y505 phosphorylation. **a** HEK 293T cells (–) or HEK 293T cells stably expressing either Lck^wt^ or Lck^Y192E^ were transiently transfected with either an empty vector (Bos) or Myc-tagged ZAP70 (75 kDa). The phosphorylation of ZAP70 on Y319 (used as a read-out for Lck activity) as well as the phosphorylation of Lck on Y505 were monitored using phosphospecific antibodies. One representative experiment is shown (n = 5). **b** Parental TCR/CD3^+^ HEK 293T cells (–) or TCR/CD3^+^ HEK 293T cells stably expressing Lck^wt^, or Lck^Y192E^ were transiently co-transfected with either a Myc-tagged ZAP70 or an empty vector (Bos). Subsequently, cells were lysed and the phosphorylation of pY319 of ZAP70 and pY505 of Lck were assessed using phosphospecific antibodies. One representative experiment is shown (n = 6). **c**, **d** The densiometric mean values of the phosphosignal pY319 in **a** and **b** were normalized against the value of the total ZAP70 signal. The bar graph shows the calculated mean + SEM values of the normalized ZAP70 phosphorylation in HEK 293T (**c**) and the TCR/CD3^+^ HEK 293T (**d**) cell systems. One dot represents one experiment. **e** Photo-caged Lck (WT, Y394F and Y192E) and ZAP70 were transiently transfected in TCR/CD3^+^ HEK 293T cells. Lck kinase activity was initiated by global illumination of the cells and the kinetics of ZAP70 phosphorylation by Lck was measured at defined time points by quantitative fluorescent Western blot analysis. Data were fit using a three-parameter logistic function and are presented as mean ± SEM (n = 3)

The data described in the previous section indicated that the signaling defects of T cells expressing the Lck^Y192E^ mutant might not exclusively be due to altered Lck^Y192E^/CD45 associations that induce hyperphosphorylation of Y505, and, consequently inactivation of Lck. To assess this possibility in more detail, we analyzed Lck functions in a cellular system that lacks the main Lck regulators, CD45 and Csk. To this end, we stably expressed either Lck^wt^ or the Lck^Y192E^ mutant in CD45^−/−^/Csk^−/−^ HEK 293T cells. Figure 6a shows that under these conditions the levels of Y505 phosphorylation of Lck^wt^ and Lck^Y192E^ were indeed comparable. Still, the Lck^Y192E^ mutant was severely impaired in phosphorylating ZAP70 on Y319 (Fig. 6a). On average, we found an approximately 40% reduction of ZAP70 phosphorylation in HEK 293T cells expressing Lck^Y192E^ (Fig. 6c). Thus, in all tested systems (i.e. mouse primary T cells, Jurkat, and HEK 293T cell lines) Lck^Y192E^ was found to be incapable of phosphorylating ZAP70. However, this appears to be independent of Y505 phosphorylation.

One disadvantage of the HEK 293T cells is that parental HEK cells lack expression of a functional TCR/CD3 complex. To circumvent this problem, we made use of a recently described HEK 293T cell variant expressing a complete human TCR (HEK-TCR cells) [29]. Again, we generated stable transfectants expressing either Lck^wt^ or the Lck^Y192E^ mutant. In both transfectants we transiently co-expressed ZAP70. Similar to the data obtained in the parental non-TCR HEK cells, the phosphorylation of ZAP70 was strongly reduced in HEK-TCR cells expressing the Lck^Y192E^ mutant (Fig. 6b, d) while the phosphorylation levels of Y505 of Lck^wt^ and the Lck^Y192E^ mutant were comparable (Fig. 6c). Of note, also in the HEK cell system the subcellular localization of LckY^192E^ was unaffected (Additional file 1: Fig. S4). Moreover, anti-CD3 staining of the HEK-TCR cells demonstrated identical plasma membrane localization of the CD3ε chain (Additional file 1: Fig. S4).

Finally, we took advantage of a photo-caged Lck in which the initiation of Lck enzymatic activity can be temporally controlled upon illumination [17]. Photo-caged Lck^Y192E^ and Lck^wt^ were expressed in HEK-TCR cells together with ZAP70. Subsequently, the rate of ZAP70 phosphorylation by Lck was measured upon illumination of the cells. In agreement with the data shown above, also in this system Lck^Y192E^ had significantly reduced ZAP70 phosphorylation kinetics when compared to Lck^wt^ (Fig. 6e). Collectively, the data from HEK 293T cells support the idea that the defective function of Lck^Y192E^ likely does not exclusively result from hyperphosphorylation of Y505.

### Altered TCR-induced interaction between the TCR/CD3 and Lck^Y192E^

We next aimed at assessing the relationship between Lck^Y192E^ and the TCR/CD3 complex. We first attempted to analyze TCR-mediated phosphorylation of TCRζ a signaling event which is located upstream of ZAP70 activation. However, we failed to see a highly reproducible reduction of the phospho-TCRζ signal upon TCR stimulation in J.Lck T cells stably re-expressing Lck^Y192E^ (Additional file 1: Fig. S5).

**Fig. 7:**
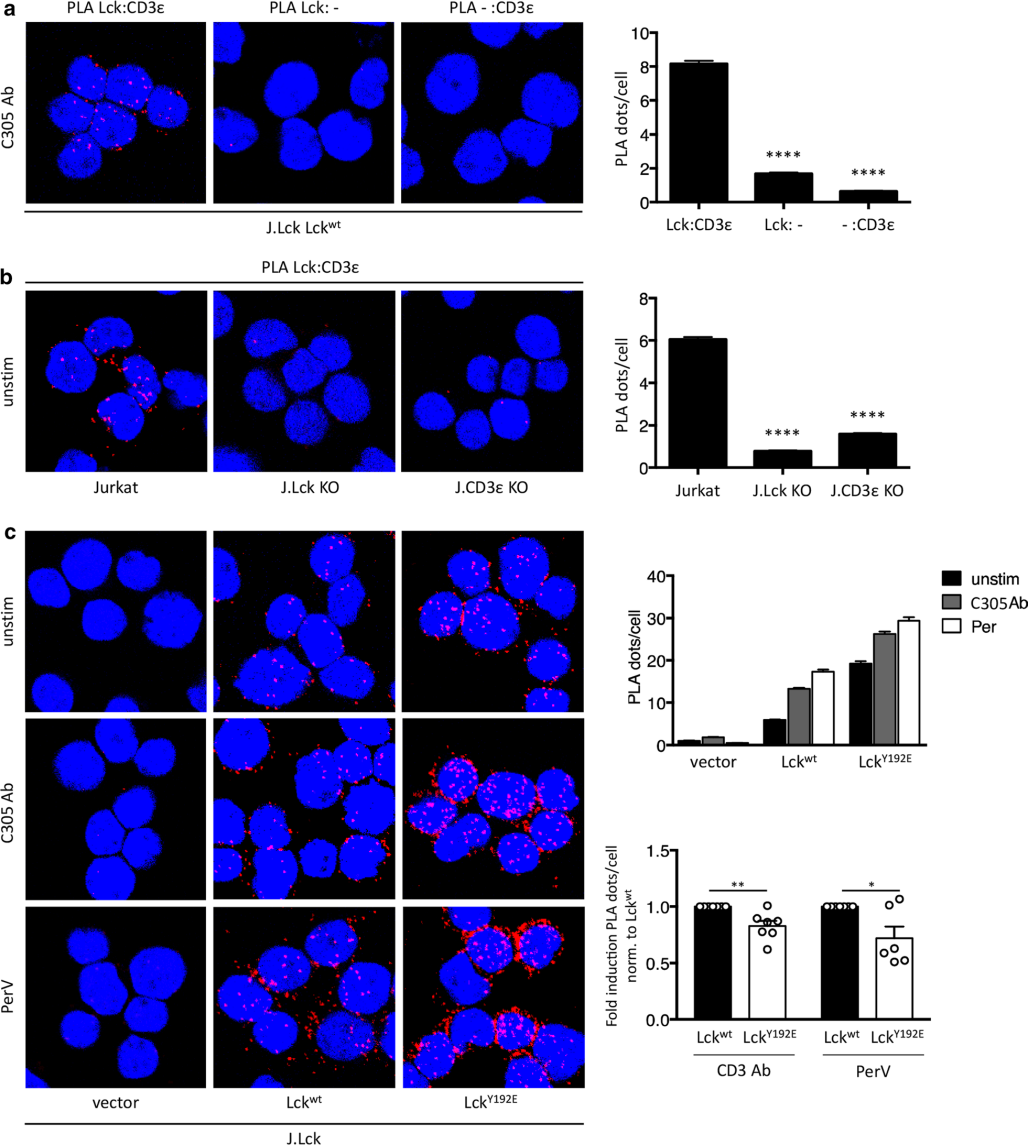
Y192 influences ligand-induced closed proximity between Lck and the TCR. **a** Technical PLA controls in J.Lck cells reconstituted with Lck^wt^ stimulated with a CD3 antibody at 37 °C for 5 min. PLA was performed with both primary antibodies (left), with only the anti-Lck primary antibody (middle) or with only the anti-CD3ε primary antibody (right). In all cases, both secondary antibodies were used. The quantification of two independent experiments was analyzed using One-way ANOVA test (bar diagram). Mean values ± SEM are shown. ****p < 0.0001. **b** Biological PLA controls in Jurkat cells expressing surface TCR and Lck (left), Jurkat cells lacking Lck expression by CRISPR/Cas9 gene editing (J.Lck, middle) and Jurkat cells lacking CD3ε by CRISPR/Cas9 gene editing (J.CD3εKO, right). The PLA was performed between the TCR (CD3ε) and Lck at 37 °C in unstimulated conditions. The quantification of two independent experiments is shown (bar diagram). Statistical analysis was performed as in **a**. **c** In situ PLA between the TCR (CD3ε) and Lck was performed; a red fluorescent signal indicates a distance between Lck and the TCR smaller than 80 nm. J.Lck cells were transfected to stably express an empty vector, Lck^wt^ or Lck^Y192E^. Cells were either left unstimulated, stimulated with a TCR antibody (C305) or stimulated with pervanadate at 37 °C for 5 min. Nuclei were stained with DAPI. Data of one representative experiment performed in technical duplicates is shown (upper bar diagram). In order to pool independently performed experiments and to normalize for Lck expression, the fold induction between unstimulated and stimulated samples per experiment was calculated (lower bar diagram). An unpaired Student’s t test on pooled data of 6–7 independent experiments was analysed. Mean values ± SEM are shown, *p < 0.1; **p < 0.01

Upon ligand engagement of the TCR/CD3, Lck binds to the TCR/CD3 [38]. Concomitantly, the CD3 and TCRζ ITAMs are exposed and phosphorylated by Lck [38, 39]. We used proximity ligation assays (PLA) to measure the proximity between Lck and CD3ε to assess the ability of Lck^wt^ or Lck^Y192E^ to interact with the TCR/CD3. To this end, stable J.Lck-transfectants re-expressing either Lck^wt^ or Lck^Y192E^ were left unstimulated or activated via the TCR/CD3 or with pervanadate as positive control. Subsequently CD3ε/Lck-PLA was performed. Figure 7 shows that, compared to Lck^wt^, the capability of Lck^Y192E^ to interact with the activated TCR is reduced. Thus, the Lck^Y192E^ mutant has partially lost its ability to interact with the activated TCR. This in turn might decrease the local Lck activity needed to optimally phosphorylate the downstream signaling proteins such as ZAP70. Together these functional properties of Lck^Y192E^ result in attenuated TCR/CD3 signaling and a failure to initiate T-cell activation.

## Discussion

In the present study, we show that, in addition to Y394 and Y505, the signaling function of Lck is regulated by a third tyrosine residue that is located at position 192 within the SH2 domain. In vivo and in vitro analyses of a phosphomimetic mutant Y192E of Lck (Lck^Y192E^) strongly suggest that phosphorylation of Y192 inhibits Lck functions. Indeed, Lck^Y192E^ knock-in mice (but not Lck^Y192F^ knock-in mice) display a strongly impaired thymic development, which is translated into a severe T-cell lymphopenia in the spleen and the lymph nodes of the Lck^Y19E^ animals. Both observations resemble the situation that is found in conventional Lck-knockout animals [5]. In addition, peripheral murine T cells carrying the Lck^Y192E^ mutation show a dramatically blunted TCR signaling, which is paralleled by a strongly impaired T-cell activation and proliferation. Similarly, Lck^Y192E^ was unable to reconstitute TCR signaling in Lck-deficient J.Lck Jurkat T cells.

Our phenotypic and functional data are in line with and extend previous observations [27] whose key finding was that phosphorylation of Y192 impairs its association with CD45. Hence, it has been proposed that loss of the Lck^Y192E^/CD45 interaction results in hyperphosphorylation of Y505 (via Csk). Hyperphosphorylated Y505 would then bind to the internal SH2 domain of Lck, thereby forcing Lck to adopt the “closed” and enzymatically inactive conformation. Consequently, T-cell activation would be blocked at the stage of TCR/CD3 phosphorylation. Indeed, we also observed that in murine splenic T cells as well as in Lck^Y192E^-reconstituted J.Lck cells the negative regulatory Y505 of Lck is hyperphosphorylated and that Lck^Y192E^ has partially lost its ability to interact with the protein tyrosine phosphatase CD45.

Classical in vitro kinase assays are a sensitive measure to assess the enzymatic activity of Lck. Employing this technique we have recently demonstrated that 20% of all Lck molecules become de novo activated during T-cell activation and that it is this fraction of Lck that induces TCR-mediated signaling [21]. To directly assess the enzymatic activity of the Lck^Y192E^ mutant, we performed in vitro kinase assays of immunoprecipitated Lck^Y192E^ using thymocytes and splenic T cells from the Lck^Y192E^ knock-in mice or Lck^Y192E^-reconstituted J.Lck cells. In contrast to the model above and despite the hyperphosphorylation of Y505, we observed in both experimental settings that the enzymatic activity of Lck^Y192E^ was similar if not even slightly higher than the enzymatic activity of Lck^wt^. These unexpected findings prompted us to investigate the conformation of Lck^Y192E^ employing the FLIM/FRET setting that we had previously used to conduct structure/function analyses of Lck in living cells [18, 21]. This approach revealed that, in line with the in vitro kinase data, an Lck^Y192E^ biosensor showed a similar (“open”) conformation as the Lck^wt^. Hence, it appears as if the Y192E mutation, despite leading to hyperphosphorylation of Y505 and loss of the Lck/CD45 interaction, would neither influence Lck basal activity nor the Lck basal conformation.

These surprising results suggested that the severe block in TCR-mediated signaling in T cells expressing Lck^Y192E^ might not exclusively be due to the hyperphosphorylation of Y505. Several groups have recently employed HEK 293T cells to assess signal transduction mechanisms, including those in T cells [17, 27, 29]. HEK cells have the advantage that they do not express many components of the T-cell signaling machinery, including the immediate Lck regulators CD45 and Csk. Using this cellular system we clearly showed that the inability of Lck^Y192E^ to phosphorylate its physiologic substrate ZAP70 is unrelated to the phosphorylation status of Y505. Indeed, in two different HEK cellular systems (expressing or not the TCR/CD3), Lck^Y192E^ was severely impaired in its ability to phosphorylate ZAP70 despite showing comparable levels of Y505 phosphorylation as Lck^wt^. Thus, the altered interaction between Lck^Y192E^ and CD45 fails to fully explain the signaling defect that is induced by Lck^Y192E^ in Jurkat T cells and in the Lck^Y192E^ knock-in mice.

Our findings lead to the question why the Lck^Y192E^ mutant cannot transduce signals in T cells or HEK cells although its enzymatic activity does not seem to be affected by the mutation. It has been previously suggested that phosphorylation of Y192 changes the specificity of the SH2 domain of Lck for its binding partners, Pyk2, Itk, SHP-1, and TSAd, which show increased binding to the Lck^Y192E^ mutant compared to Lck^wt^ [24]. However, proteomics approaches using our stable Lck^Y192E^ Jurkat transfectants or peripheral T cells obtained from Lck^Y192E^ mutant mice so far failed to reveal data supporting that Lck^wt^ and Lck^Y192E^ form different protein complexes in T cells (Additional file 2: Table S1). Likewise, the confocal analysis of both Lck^Y192E^ expressing Jurkat and HEK cells ruled out altered subcellular localization of Lck^Y192E^.

An impaired de novo activation of Lck^Y192E^ following TCR stimulation could be responsible for the observed signaling defects. Indeed, our observation that an Lck-biosensor carrying the Lck^Y192E^ mutation does not properly open in response to CD3-mediated stimuli point into this direction. Intriguingly, a recent report has shown that Lck binds to a RK-motif within CD3ε that is exposed upon ligand-binding to the TCR [28]. This interaction results in local augmentation of Lck activity, thereby promoting TCR-mediated stimuli. Conversely, mutation of the RK-motif results in impaired T-cell activation, both in vitro and in vivo. It was therefore tempting to speculate that the Lck^Y192E^ mutant has partially lost its flexibility and thereby, the ability to interact with its natural interactions partners including CD45 and CD3ε. In line with this idea, PLA-experiments in J.Lck cells reconstituted with either Lck^wt^ or Lck^Y192E^ revealed an impaired ability of Lck^Y192E^ to associate with the activated TCR.

Taken together, our data suggest that an altered Lck/CD45 interaction is not the exclusive reason why T cells cannot develop in Lck^Y192E^ knock-in mice. We rather propose that phosphorylation of Y192 regulates the very first steps of T-cell activation by at least two complementary mechanisms: by preventing Lck association to CD45 and by hindering ligand-induced recruitment of Lck to the TCR.

## Supplementary information

**Additional file 1: Figure S1.** Normal T-cell development and T-cell activation in Lck^Y192F^ knock-in mice. (A) Thymocytes from Lck^wt^ and Lck^Y192F^ knock-in mice were stained with CD4 and CD8 antibodies. Representative dot plots from 3 independent experiments show the distribution of thymocyte subsets. Total thymocyte numbers are indicated above the dot plots. (B) Analysis of the distribution of thymic subpopulations from 3 Lck^wt^ and 3 Lck^Y192F^ knock-in mice. Statistical analyses were performed using an unpaired Student’s t test, ns = not statistically significant. (C) Peripheral T cells numbers from 4 Lck^wt^ and 4 Lck^Y192F^ knock-in mice were calculated. (D) Splenic T cells from Lck^wt^ and Lck^Y192F^ knock-in mice were stimulated with a CD3 antibody. At the indicated time points after stimulation, cells were lysed and the levels of global protein tyrosine phosphorylation and Lck expression were assessed using a pan phosphotyrosine antibody (pY total) and a Lck Ab (Lck total), respectively. One representative of 3 independent experiments is shown. Equal protein loading was verified using antibodies directed against β-actin. **Figure S2.** T-cell subsets in peripheral lymphoid organs from Lck^Y192E^ knock-in mice. (A) Lymph node (LN) (left panel) and splenic cells (right panel) from Lck^wt^ and Lck^Y192E^ mice were isolated and stained with CD4/CD44 or CD8/CD44 antibodies and analyzed by flow cytometry. Subsequently, total cell numbers of CD4^+^/CD44^low^, CD4^+^/CD44^high^, CD8^+^/CD44^low^, and CD8^+^/CD44^high^ T cells were calculated. Each dot represents one mouse. (B) Histograms show CD3 expression levels from lymph node (left panel) and spleen (right panel). The dotted line indicates Lck^Y192E^ mice. One representative histogram from 3 independent experiments is shown. (C) Cells isolated from lymph nodes and spleens were stained with a B220 antibody and analyzed by flow cytometry to identify B cells. Subsequently, absolute cell numbers were calculated. Each dot represents one mouse. Statistical analyses were performed using an unpaired Student’s t test, ****p < 0.0001, ***p < 0.001. **Figure S3.** TCR-mediated signaling in J.Lck cells cells stably reconstituted with Lck^Y192E^. (A) Jurkat T cells (JE6) and Lck-deficient Jurkat cells (J.Lck) stably expressing an empty vector, Lck^wt^, or Lck^Y192E^ were stimulated with a TCR antibody (clone: C305). After stimulation, cells were lysed and the levels of global protein tyrosine phosphorylation were assessed using a pan phosphotyrosine antibody (pY total, clone 4G10) and phosphospecific antibodies against ZAP70. Lck expression and equal protein loading were verified using antibodies directed against total Lck and β-actin, respectively. One representative of 2 independent experiments is shown. Lanes 5/6 versus 7/8 display Lck^wt^-reconstituted J.Lck cells expressing different amounts of Lck. (B) The histogram shows CD3 expression from J.Lck stably expressing Lck^wt^ and Lck^Y192E^. **Figure S4.** Subcellular localization of Lck^Y192E^ and CD3ε in HEK293T and HEK293T + TCR/CD3. (A, B) Fluorescence (left panel) and Brightfield (BF, right panel) pictures of HEK 293T cells stably expressing either Lck^wt^ (A) or Lck^Y192E^ (B) were labeled with an Lck antibody and a secondary antibody with the fluorophore dylight 649. (C, D) Fluorescence (left panel) and Brightfield (BF, right panel) pictures of TCR/CD3^+^ HEK 293T cells stably expressing Lck^wt^ (C) or Lck^Y192E^ (D) were stained with CD3ε antibody and a secondary antibody tagged with dylight 649. (E, F) TCR/CD3 + HEK 293T cells stably expressing Lck^wt^ E) or Lck^Y192E^ (F) were stained with a Lck antibody and a secondary antibody coupled with dylight 649. **Figure S5.** TCR/CD3-mediated phosphorylation of CD3ζ in J.Lck cells reconstituted with Lck^wt^ or Lck^Y192E^. Data of four independent experiments in which the TCR-mediated induction of phosphorylation of TCRζ and Y319 of Zap70 was determined. The blot corresponding to experiment 1 also shows intra-experimental variations (for Lck^wt^). Besides the phosphorylation of TCRζ and pY319 of Zap70 the expression of Lck was determined. Anti-CD3ε staining was used as loading control. The molecular weight markers shown on the left correspond to the blots of experiment 1.

**Additional file 2: Table S1.** Mass spec data of Lck deficient Jurkat T cells (J.CaM 1.6) stably expressing Lck^wt^ and Lck^Y192E^.

## Data Availability

The datasets used and/or analysed during the current study are available from the corresponding author on reasonable request.
