# Supplementary figures and images for: Tyrosine 192 within the SH2 domain of the Src-protein tyrosine kinase p56Lck regulates T-cell activation independently of Lck/CD45 interactions
Source: Cell Commun Signal. 2020 Nov 23;18:183. doi: 10.1186/s12964-020-00673-z (PMC7682018; doi:10.1186/s12964-020-00673-z)

# Supplementary Figures

Fig. S1:

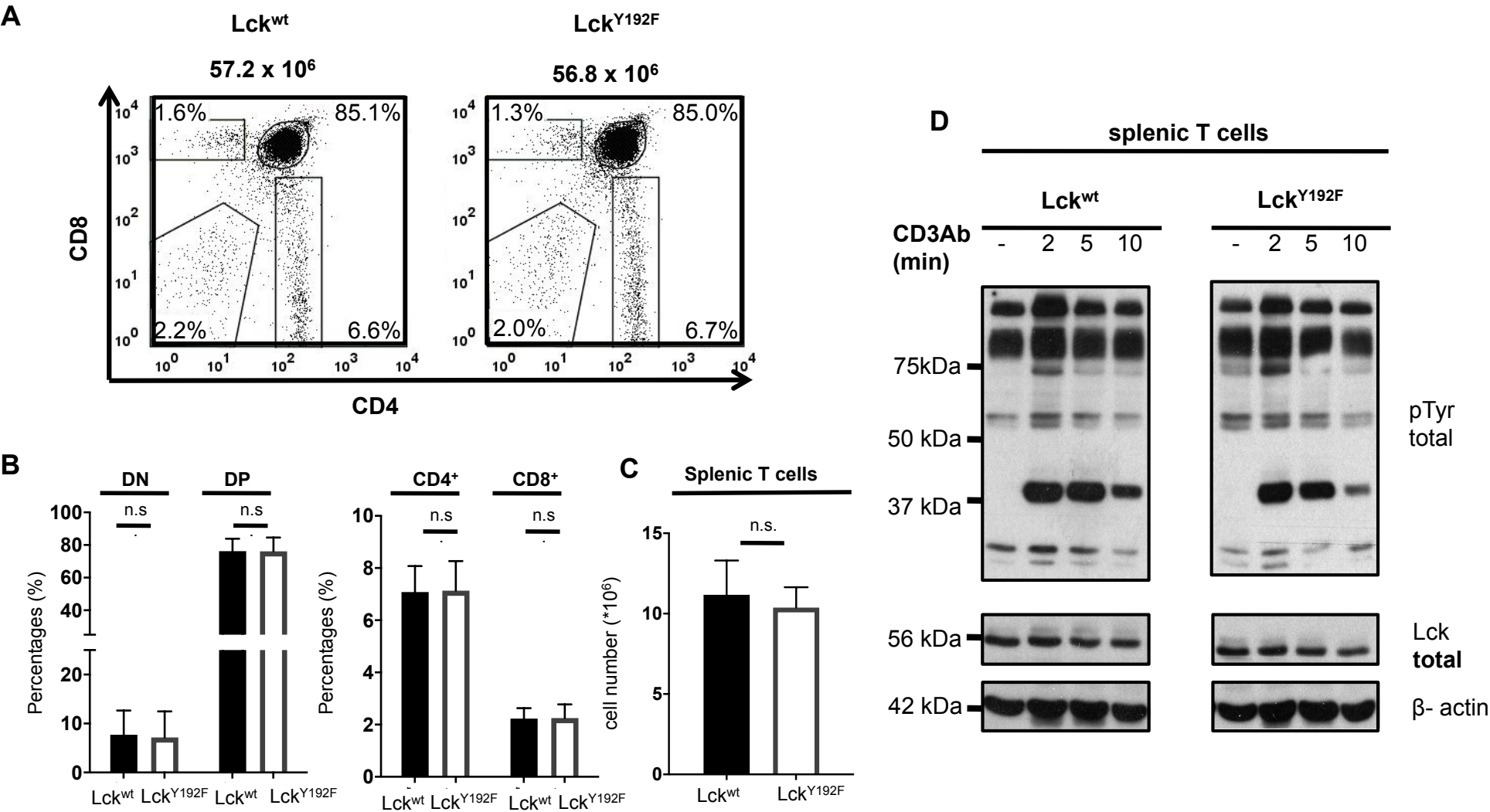

Fig. S2:

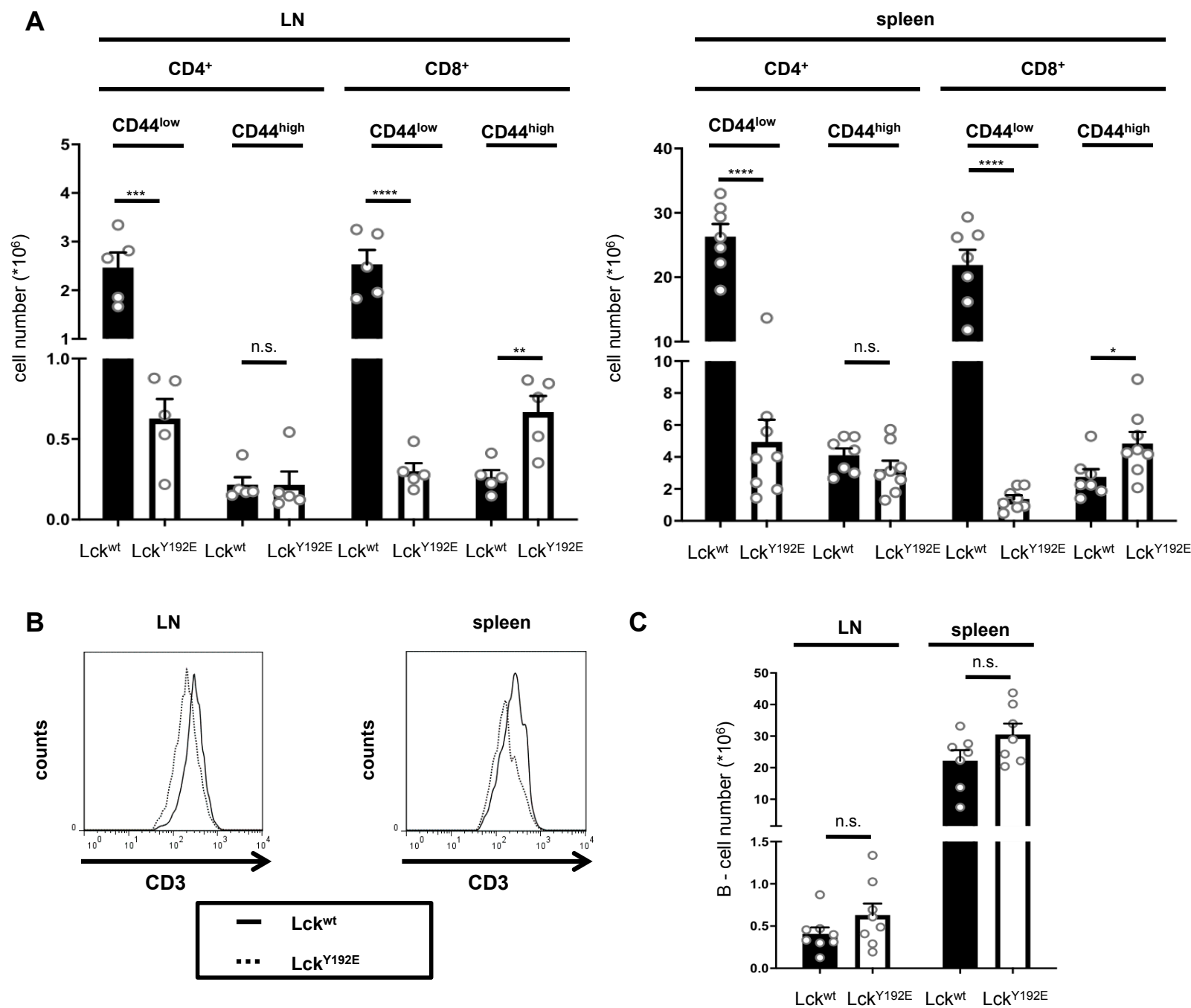

Fig. S3:

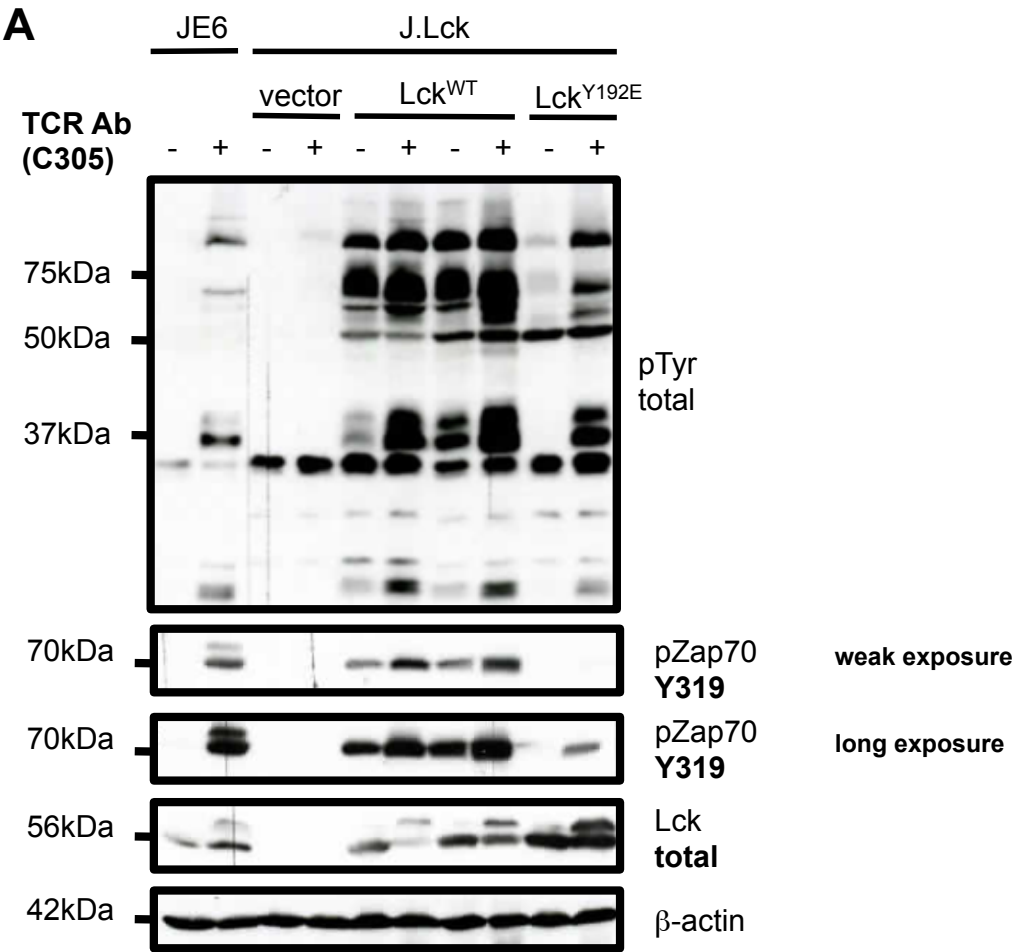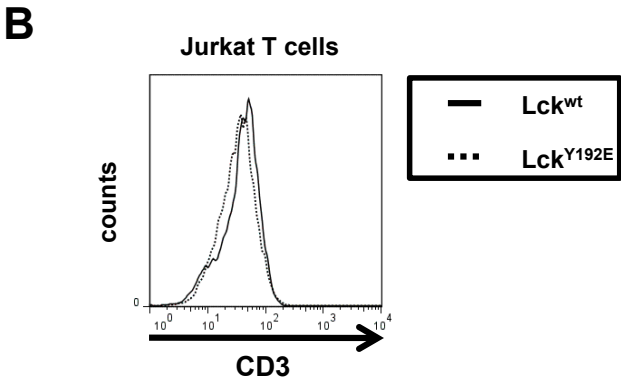

**Fig. S4:**

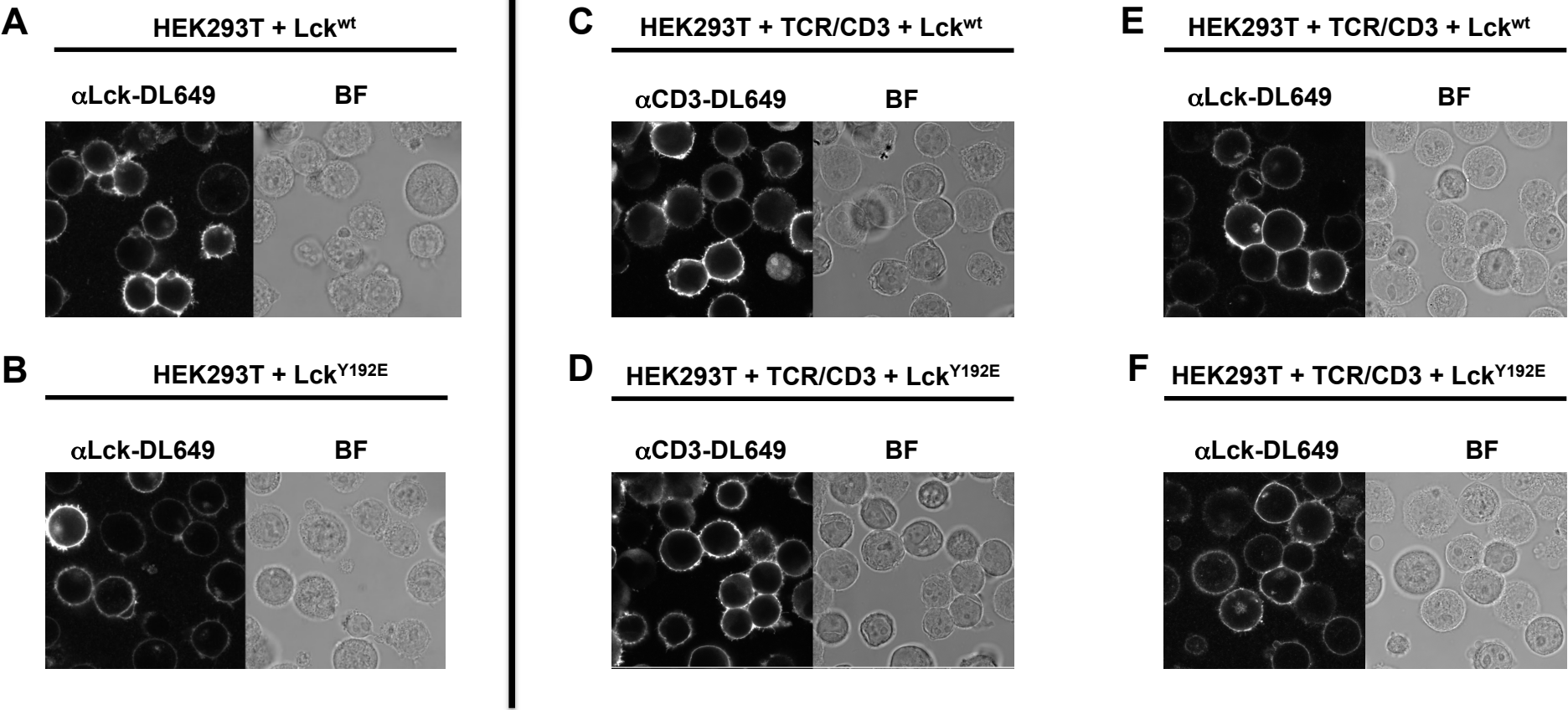

**Fig. S5:**

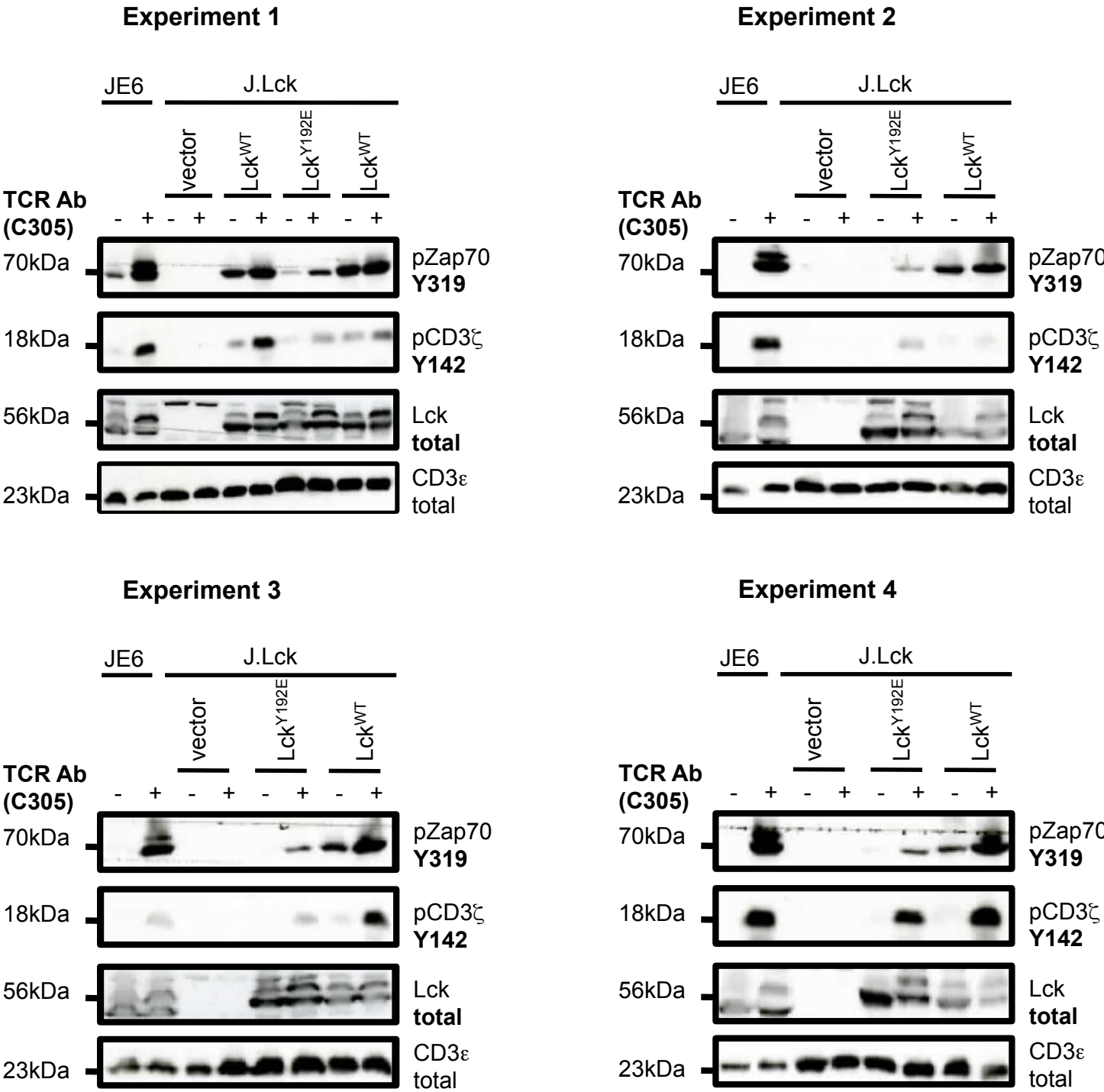

Supplement: Supplementary file 2 — Additional file 1: Figure S1. Normal T-cell development and T-cell activation in LckY192F knock-in mice. (A) Thymocytes from Lckwt and LckY192F knock-in mice were stained with CD4 and CD8 antibodies. Representative dot plots from 3 independent experiments show the distribution of thymocyte subsets. Total thymocyte numbers are indicated above the dot plots. (B) Analysis of the distribution of thymic subpopulations from 3 Lckwt and 3 LckY192F knock-in mice. Statistical analyses were performed using an unpaired Student’s t test, ns = not statistically significant. (C) Peripheral T cells numbers from 4 Lckwt and 4 LckY192F knock-in mice were calculated. (D) Splenic T cells from Lckwt and LckY192F knock-in mice were stimulated with a CD3 antibody. At the indicated time points after stimulation, cells were lysed and the levels of global protein tyrosine phosphorylation and Lck expression were assessed using a pan phosphotyrosine antibody (pY total) and a Lck Ab (Lck total), respectively. One representative of 3 independent experiments is shown. Equal protein loading was verified using antibodies directed against β-actin. Figure S2. T-cell subsets in peripheral lymphoid organs from LckY192E knock-in mice. (A) Lymph node (LN) (left panel) and splenic cells (right panel) from Lckwt and LckY192E mice were isolated and stained with CD4/CD44 or CD8/CD44 antibodies and analyzed by flow cytometry. Subsequently, total cell numbers of CD4+/CD44low, CD4+/CD44high, CD8+/CD44low, and CD8+/CD44high T cells were calculated. Each dot represents one mouse. (B) Histograms show CD3 expression levels from lymph node (left panel) and spleen (right panel). The dotted line indicates LckY192E mice. One representative histogram from 3 independent experiments is shown. (C) Cells isolated from lymph nodes and spleens were stained with a B220 antibody and analyzed by flow cytometry to identify B cells. Subsequently, absolute cell numbers were calculated. Each dot represents one mouse. Statistical an [file 12964_2020_673_MOESM2_ESM.pdf]
