# Supplementary material for: Tyrosine 192 within the SH2 domain of the Src-protein tyrosine kinase p56Lck regulates T-cell activation independently of Lck/CD45 interactions
Source: Cell Commun Signal. 2020 Nov 23;18:183. doi: 10.1186/s12964-020-00673-z (PMC7682018; doi:10.1186/s12964-020-00673-z)
Supplement: Supplementary file 3 — Additional file 2: Table S1. Mass spec data of Lck deficient Jurkat T cells (J.CaM 1.6) stably expressing Lckwt and LckY192E. [file 12964_2020_673_MOESM3_ESM.docx]

**Additional file 2: Table S1.** Mass spec data of Lck deficient Jurkat T cells (J.CaM 1.6) stably expressing Lck^wt^ and Lck^Y192E^

Lck was immunoprecipitated from Lck deficient Jurkat T cells (J.CaM 1.6) stably expressing either Lck^wt^ or Lck^Y192E^. To assess differences in the protein interactions of the Lck^Y192E^ mutant, mass spectrometry analysis was performed.

| **Accession** | **Description** | **found in Lck^wt^** |  |  | **found in Lck^Y192E^** |  |  |
| --- | --- | --- | --- | --- | --- | --- | --- |
|  |  | in n of 3 experiments | **mean (-10lgP)** | **mean (number of peptides)** | in n of 3 experiments | **mean (-10lgP)** | **mean (number of peptides)** |
| P06239\|LCK_HUMAN | Tyrosine-protein kinase Lck | 3 # 3 | 341.3933333 | 20 | 3 # 3 | 334.9233333 | 20.66666667 |
| Q9Y608\|LRRF2_HUMAN | Leucine-rich repeat flightless-interacting protein 2 | 2 # 3 | 172.91 | 5.5 | 2 # 3 | 149.365 | 5 |
| Q9Y266\|NUDC_HUMAN | Nuclear migration protein nudC | 2 # 3 | 131.99 | 3 | 2 # 3 | 139.07 | 4 |
| Q9UQ35\|SRRM2_HUMAN | Serine/arginine repetitive matrix protein 2 | 2 # 3 | 109.59 | 5.5 | 2 # 3 | 71.405 | 2 |
| Q9ULV4\|COR1C_HUMAN | Coronin-1C | 2 # 3 | 262.825 | 9.5 | 2 # 3 | 251.625 | 11 |
| Q9UKX3\|MYH13_HUMAN | Myosin-13 | 3 # 3 | 143.345 | 7 | 3 # 3 | 154.69 | 5.5 |
| Q9NYL9\|TMOD3_HUMAN | Tropomodulin-3 | 2 # 3 | 230.89 | 8 | 2 # 3 | 174.26 | 5 |
| Q9BUA6\|MYL10_HUMAN | Myosin regulatory light chain 10 | 2 # 3 | 91.565 | 2.5 | 2 # 3 | 102.36 | 2.5 |
| Q7Z406\|MYH14_HUMAN | Myosin-14 | 2 # 3 | 215.83 | 14.5 | 2 # 3 | 227.58 | 12.5 |
| Q6P996\|PDXD1_HUMAN | Pyridoxal-dependent decarboxylase domain-containing protein 1 | 2 # 3 | 95.505 | 2.5 | 2 # 3 | 73.15 | 3 |
| Q562R1\|ACTBL_HUMAN | Beta-actin-like protein 2 | 2 # 3 | 171.52 | 7 | 2 # 3 | 151.715 | 6 |
| Q13045\|FLII_HUMAN | Protein flightless-1 homolog | 2 # 3 | 105.28 | 3.5 | 2 # 3 | 114.57 | 5 |
| Q07955\|SRSF1_HUMAN | Serine/arginine-rich splicing factor 1 | 2 # 3 | 105.52 | 1.5 | 2 # 3 | 115.82 | 2.5 |
| P62805\|H4_HUMAN | Histone H4 | 3 # 3 | 146.89 | 5.666666667 | 3 # 3 | 109.2733333 | 2.333333333 |
| P61160\|ARP2_HUMAN | Actin-related protein 2 OS=Homo sapiens OX=9606 GN=ACTR2 PE=1 SV=1 | 2 # 3 | 217.875 | 6.5 | 2 # 3 | 191.58 | 5 |
| P61158\|ARP3_HUMAN | Actin-related protein 3 OS=Homo sapiens | 2 # 3 | 211.11 | 6 | 2 # 3 | 217.74 | 9 |
| P60660\|MYL6_HUMAN | Myosin light polypeptide 6 | 3 # 3 | 261.645 | 11.5 | 3 # 3 | 251.68 | 11.5 |
| P52907\|CAZA1_HUMAN | F-actin-capping protein subunit alpha 1 | 2 # 3 | 200.765 | 6 | 2 # 3 | 209.275 | 6.5 |
| P47756\|CAPZB_HUMAN | F-actin-capping protein subunit beta | 2 # 3 | 138.7 | 3 | 2 # 3 | 133.625 | 3 |
| P45880\|VDAC2_HUMAN | Voltage-dependent anion-selective channel protein 2 | 2 # 3 | 124.165 | 2 | 2 # 3 | 116.81 | 2 |
| P35749\|MYH11_HUMAN | Myosin-11 | 2 # 3 | 293.675 | 20 | 2 # 3 | 299.015 | 21.5 |
| P35580\|MYH10_HUMAN | Myosin-10 | 2 # 3 | 513.73 | 85.5 | 2 # 3 | 500.355 | 81.5 |
| P19474\|RO52_HUMAN | E3 ubiquitin-protein ligase TRIM21 | 3 # 3 | 214.17 | 8.333333333 | 3 # 3 | 214.6366667 | 9 |
| P16989\|YBOX3_HUMAN | Y-box-binding protein 3 | 2 # 3 | 102.315 | 2 | 2 # 3 | 71.64 | 3 |
| P11142\|HSP7C_HUMAN | Heat shock cognate 71 kDa protein | 2 # 3 | 179.54 | 5.5 | 2 # 3 | 153.315 | 4 |
| P10515\|ODP2_HUMAN | Dihydrolipoyllysine-residue acetyltransferase component of pyruvate dehydrogenase complex  mitochondrial | 2 # 3 | 204 | 6.5 | 2 # 3 | 182.275 | 6 |
| P0DP25\|CALM3_HUMAN | Calmodulin-3 | 2 # 3 | 232.925 | 9 | 2 # 3 | 243.77 | 8 |
| P0DP24\|CALM2_HUMAN | Calmodulin-2 | 2 # 3 | 232.925 | 9 | 2 # 3 | 243.77 | 8 |
| P0DP23\|CALM1_HUMAN | Calmodulin-1 | 2 # 3 | 232.925 | 9 | 2 # 3 | 243.77 | 8 |
| P08670\|VIME_HUMAN | Vimentin | 3 # 3 | 267.73 | 12.3 | 3 # 3 | 203.34 | 10.6 |
| P06753\|TPM3_HUMAN | Tropomyosin alpha-3 chain O | 2 # 3 | 174.045 | 7.5 | 2 # 3 | 162.96 | 7.5 |
| O43795\|MYO1B_HUMAN | Unconventional myosin-Ib | 2 # 3 | 260.35 | 12.5 | 2 # 3 | 247.53 | 10.5 |
| O15144\|ARPC2_HUMAN | Actin-related protein 2/3 complex subunit 2 | 2 # 3 | 160.715 | 2 | 2 # 3 | 162.01 | 3 |
| B0I1T2\|MYO1G_HUMAN | Unconventional myosin-Ig | 2 # 3 | 394.89 | 32 | 2 # 3 | 382.515 | 31.5 |
| P67936\|TPM4_HUMAN | Tropomyosin alpha-4 chain | 2 # 3 | 223.345 | 12 | 2 # 3 | 202.08 | 12 |
| O95405\|ZFYV9_HUMAN | Zinc finger FYVE domain-containing protein 9 | 2 # 3 | 30.17 | 1.5 | 2 # 3 | 26.79 | 1 |
| Q00975\|CAC1B_HUMAN | Voltage-dependent N-type calcium channel subunit alpha-1B | 2 # 3 | 29.205 | 1.5 | 0 # 3 |  |  |
| Q07020\|RL18_HUMAN | 60S ribosomal protein L18 | 3 # 3 | 91.83333333 | 1.333333333 | 0 # 3 |  |  |
| Q5VST9\|OBSCN_HUMAN | Obscurin OS=Homo sapiens | 3 # 3 | 33.02666667 | 2.3333333 | 0 # 3 |  |  |
| Q9UKV3\|ACINU_HUMAN | Apoptotic chromatin condensation inducer in the nucleus | 2 # 3 | 93.06 | 2 | 0 # 3 |  |  |
| P33778\|H2B1B_HUMAN | Histone H2B type 1-B OS=Homo sapiens | 0 # 3 |  |  | 2 # 3 | 80.23 | 2 |
| P63261\|ACTG_HUMAN | Actin  cytoplasmic 2 OS=Homo sapiens | 0 # 3 |  |  | 2 # 3 | 275.685 | 24 |
| Q9Y5S9\|RBM8A_HUMAN | RNA-binding protein 8A | 0 # 3 |  |  | 2 # 3 | 53.52 | 1 |
| Q9Y4D7\|PLXD1_HUMAN | Plexin-D1 | 0 # 3 |  |  | 2 # 3 | 22.46 | 1 |
| Q9P2E9\|RRBP1_HUMAN | Ribosome-binding protein 1 | 0 # 3 |  |  | 2 # 3 | 27.635 | 1.5 |
| Q92922\|SMRC1_HUMAN | SWI/SNF complex subunit SMARCC1 | 0 # 3 |  |  | 2 # 3 | 102.895 | 2 |
